# Areas of Interest and Social Consideration of Antidepressants on English Tweets: A Natural Language Processing Classification Study

**DOI:** 10.3390/jpm12020155

**Published:** 2022-01-25

**Authors:** Laura de Anta, Miguel Angel Alvarez-Mon, Miguel A. Ortega, Cristina Salazar, Carolina Donat-Vargas, Javier Santoma-Vilaclara, Maria Martin-Martinez, Guillermo Lahera, Luis Gutierrez-Rojas, Roberto Rodriguez-Jimenez, Javier Quintero, Melchor Alvarez-Mon

**Affiliations:** 1 Department of Psychiatry and Mental Health, Hospital Universitario Infanta Leonor, 28031 Madrid, Spain; lanta.79@gmail.com (L.d.A.); marymm37@icloud.com (M.M.-M.); fjquinterog@salud.madrid.org (J.Q.); 2 Department of Medicine and Medical Specialities, Faculty of Medicine and Health Sciences, University of Alcalá, 28801 Alcalá de Henares, Spain; guillermo.lahera@gmail.com (G.L.); mademons@gmail.com (M.A.-M.); 3 Ramón y Cajal Health Research Institute (IRYCIS), 28034 Madrid, Spain; 4 Departamento Teoría de la Señal y Comunicaciones y Sistemas Telemáticos y Computación, Escuela Técnica Superior de Ingeniería de Telecomunicación, Universidad Rey Juan Carlos, 28942 Fuenlabrada, Spain; cris20salazar@gmail.com; 5 Unit of Cardiovascular and Nutritional Epidemiology, Institute of Environmental Medicine (IMM), Karolinska Institute, 171 77 Stockholm, Sweden; carolina.donat.vargas@ki.se; 6 IBM Data and AI Expert Labs and Learning, London WC2N 5DU, UK; jasantovi@gmail.com; 7 CIBERSAM (Biomedical Research Networking Centre in Mental Health), 22807 Madrid, Spain; roberto.rodriguez.jimenez@gmail.com; 8 Psychiatry Service, Príncipe de Asturias University Hospital, 28805 Alcalá de Henares, Spain; 9 Psychiatry Service, Hospital Clínico San Cecilio, 18016 Granada, Spain; gutierrezrojasl@hotmail.com; 10 Instituto de Investigación Sanitaria Hospital 12 de Octubre (imas 12), Universidad Complutense de Madrid (UCM), 28040 Madrid, Spain; 11 Immune System Diseases-Rheumatology and Oncology Service, University Hospital Príncipe de Asturias, CIBEREHD, 28805 Alcalá de Henares, Spain

**Keywords:** depression, psychopharmacology, antidepressants, machine learning, artificial intelligence, pharmacoepidemiology

## Abstract

Background: Antidepressants are the foundation of the treatment of major depressive disorders. Despite the scientific evidence, there is still a sustained debate and concern about the efficacy of antidepressants, with widely differing opinions among the population about their positive and negative effects, which may condition people’s attitudes towards such treatments. Our aim is to investigate Twitter posts about antidepressants in order to have a better understanding of the social consideration of antidepressants. Methods: We gathered public tweets mentioning antidepressants written in English, published throughout a 22-month period, between 1 January 2019 and 31 October 2020. We analysed the content of each tweet, determining in the first place whether they included medical aspects or not. Those with medical content were classified into four categories: general aspects, such as quality of life or mood, sleep-related conditions, appetite/weight issues and aspects around somatic alterations. In non-medical tweets, we distinguished three categories: commercial nature (including all economic activity, drug promotion, education or outreach), help request/offer, and drug trivialization. In addition, users were arranged into three categories according to their nature: patients and relatives, caregivers, and interactions between Twitter users. Finally, we identified the most mentioned antidepressants, including the number of retweets and likes, which allowed us to measure the impact among Twitter users. Results: The activity in Twitter concerning antidepressants is mainly focused on the effects these drugs may have on certain health-related areas, specifically sleep (20.87%) and appetite/weight (8.95%). Patients and relatives are the type of user that most frequently posts tweets with medical content (65.2%, specifically 80% when referencing sleep and 78.6% in the case of appetite/weight), whereas they are responsible for only 2.9% of tweets with non-medical content. Among tweets classified as non-medical in this study, the most common subject was drug trivialization (66.86%). Caregivers barely have any presence in conversations in Twitter about antidepressants (3.5%). However, their tweets rose more interest among other users, with a ratio 11.93 times higher than those posted by patients and their friends and family. Mirtazapine is the most mentioned antidepressant in Twitter (45.43%), with a significant difference with the rest, agomelatine (11.11%). Conclusions: This study shows that Twitter users that take antidepressants, or their friends and family, use social media to share medical information about antidepressants. However, other users that do not talk about antidepressants from a personal or close experience, frequently do so in a stigmatizing manner, by trivializing them. Our study also brings to light the scarce presence of caregivers in Twitter.

## 1. Introduction

Depression is a psychiatric illness characterized by sadness, loss of interest or pleasure, sense of guilt and/or low self-esteem, sleep and/or eating disorders, fatigue and lack of concentration [1]. In its most severe type, depression can lead to suicide and to a higher risk of mortality [2,3,4]. Depression usually has a chronic course and is detrimental to work potential and the quality of life of the individual [5,6,7]. It is one of the most prevalent disorders at a worldwide level [8,9]. It affects more than 300 million people in the world and is one of the main causes of disability [10]. It is responsible for 4.3% of morbidity worldwide [11]. The current known prevalence will most likely increase in the near future due to the COVID-19 pandemic [12,13,14]. Although evidence-based psychosocial intervention along with pharmacotherapy can help patients get better by reducing their symptoms, it is of great importance to keep expanding the knowledge and comprehension of these treatments in order to tackle the increasing demand.

Antidepressants are the foundation of the treatment of major depressive disorders. These, organised into several drug types with different mechanisms of action, are widely used and are available worldwide. Meta-analysis of wide renown has proven their efficacy against placebo, whereas psychotherapeutic treatment is the alternative type of treatment for milder cases [15,16,17]. Despite this, there is still a sustained debate and concern about the efficacy of antidepressants, as studies demonstrate that short-term benefits are moderate and long-term benefits studies are scarce [18,19,20]. In this line of thought, interpersonal variability is also of influence and, at the moment, there are no biological markers to assess the most beneficial drug for each individual [15,21]. Despite the meta-analysis, existing evaluations of both negative and positive effects of antidepressants are based in group data and can only be used for individual recommendations [22].

A measure of antidepressants’ efficacy has been normally assessed with randomised controlled trials and databases, which are both liable to bias [15,23]. On the other hand, patients’ personal beliefs, that play a significant role in different aspects related to pharmacological treatment, have traditionally been studied using qualitative and arduous methods, which needed an update. On this regard, in recent years, research analysing social media posts has been gathering more interest as a tool leading to a more thorough comprehension of patient’s beliefs, and of other factors that are involved in both their illness experience and therapeutic process [24,25,26,27]. These analyses also enable a better understanding of the views of non-patient individuals and include those of patients reluctant to go to a healthcare professional [26,27,28]. It is also noteworthy that conversations in social media take place in a more informal and spontaneous environment than the ones that take place during an appointment with a professional, so they are more likely to show true beliefs [29,30,31].

People with mental illness appear to use social media to share their illness experience or to seek advice from people with a similar ailment [32]. In the case of depression, we have to take into account that patients with a depressive disorder are more prone to social isolation and tend to not seek help [33]. Previous research shows that social media enables a supportive environment to discuss depression [34]. It is not known if people suffering from depression talk about their experience with antidepressants in social media, although some research about different aspects of the antidepressant treatment has already been conducted [35]. Along this line, the aims of our study can be summarized as follows: (1) assess the frequency of on-line communication about antidepressants in Twitter; (2) portray the type of user that participates in these conversations; (3) identify the main content of tweets and the interest they rise; and (4) analyse mentions to specific antidepressants.

## 2. Materials and Methods

### 2.1. Search Strategy and Data Collection in Twitter

In this study, we focused on searching tweets that referenced antidepressants. We gathered all tweets using the following list of keywords: agomelatine, Valdoxan, mirtazapine, rexer, afloyan, remeron, trazodone, deprax, amitriptyline, tryptizole, clomipramine, anafranil, imipramine, tofranil, nortriptyline, paxtibi, pamelor, bupropion, elontril, zyntabac, wellbutrin, zyban, desvenlafaxine, pristiq, venlafaxine, vandral, vandral retard, dobupal, dobupal retard, effexor xr, duloxetine, cymbalta, xeristar, vortioxetine, brintellix, reboxetine, irenor, citalopram, prisdal, seropram, celexa, escitalopram, cipralex, esertia, lexapro, fluoxetine, prozac, adofen, sarafem, paroxetine, motivan, seroxat, daparox, paxil, sertraline, aremis, besitran, zoloft. We organised them into eighteen groups depending on their active ingredient.

The inclusion criteria of tweets were: (1) Being public; (2) Usage of the aforementioned keywords; (3) Posted between the 1st of January 2019 and the 31st of October 2020; (4) Written in English. We chose this twenty-two-month period to avoid any possible bias in their content. The main goal was to bypass special circumstances both within the health field (such as the publishing of relevant scientific papers about one of the antidepressants included in our study, references to congresses or workshops, etc.) and not health related (such as seasons, a post about this type of drug by a famous person, the premiere of a movie or TV series about antidepressants, etc.) that may have influenced the content.

We also registered the number or retweets and likes that each tweet got as an indicator of the existing interest about a particular subject, the date and hour of each tweet, a permanent link to the tweet and the description of the profile of each user [36]. Tweet Binder, the search engine we used, allows access to the 100% of the public tweets that match specific criteria.

### 2.2. Content Analysis Process

**Figure 1:**
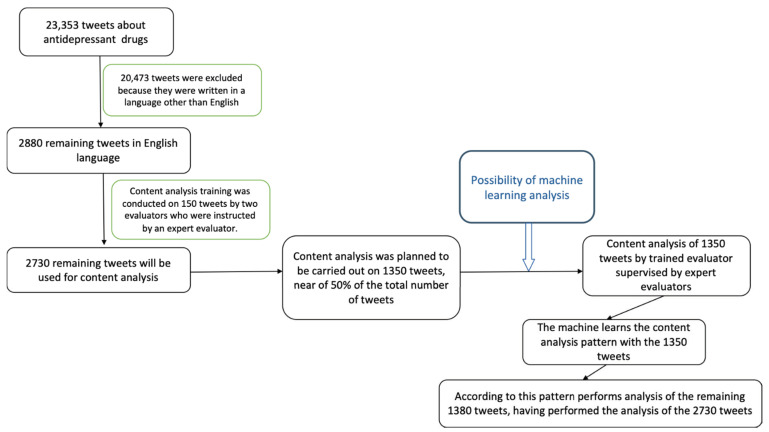
Flowchart of data management.

Using the aforementioned keywords, the search engine retrieved 23,353 tweets. After filtering according to our inclusion criteria, we ended with 2880 tweets to take into consideration for the content analysis. We randomly selected 1500 for being manually analysed (Figure 1).

After this, we drafted a codebook based on our study approach, our previous experience in the analysis of tweets and what we determined to be the most prevalent topics. Finally, our team, LAT and MAAM, individually and manually analysed 150 tweets out of the 1500 previously randomised set, in order to test the suitability of the codebook. The discrepancies were discussed with a senior author (MAM), and after reviewing the codebook, we manually coded the remaining 1350 tweets.

During the content analysis we first considered if tweets were deemed unclassifiable or classifiable. On a first approach every tweet considered classifiable was sorted depending on its content into medical or non-medical. The ones with a medical content, were organised into the following categories, according to their area of clinical interest: general aspects (including aspects such as quality of life, mood, anxiety, cognitive complaint and sexual dysfunction), sleep, appetite/weight, and somatic complaints. If it could not be rendered into any of these areas of interest, the tweet was regarded as undetermined. On the other hand, tweets of non-medical content were classified into three categories: commercial nature (including all economic activity, drug promotion, education or outreach), help request/offer, and drug trivialization. Tweets not falling into any of these categories were classified as non-specific. In addition, we also took into consideration the following aspects: whether the tweet made reference to a psychiatric diagnosis, if what is mentioned about the drug is scientifically accurate, or whether a medical prescription is stated. Finally, we arranged the type of user into 3 categories: patient and friends/relatives, caregivers (both professional and institutions), and interaction between Twitter users (those tweets mentioning other users, @username). In cases where it was not possible to know the nature of the user, it was considered undetermined. Examples of tweets are shown in Supplementary Materials File S1.

### 2.3. Natural Language Processing Classifier

The total number of tweets that was selected after filtering was 2880, and out of them 1500 were manually classified and tagged and used to train, test and validate Machine Learning classifiers for each of the categories. These classifiers were then applied to the remaining universe of tweets (1380), the not classified ones, to complete the classification and labelling of all the targeted tweets. To train the classifiers, a transformer English model BERT was used and the library to deploy it was “k-train” [37,38,39]. The transformers BERT model had understanding of standard English as it had been trained on a large corpus of English language and was suitable for tweets as they were also written in standard English. This transformer model was used as a base model to be trained as a classifier with the labelled tweets for each of the categories, this is transfer learning as the BERT classifier’s general understanding was then tailored to a specific task. This produced a classifier for each category that could then be applied to the tweets.

The following additional features were generated to improve the understanding of the selected set and during the data exploration phase: number of tokens that the sentence contained, total length of the Tweet in characters, language of the tweet and the extracted hashtags from the tweet. Moreover, to improve the Machine Learning classifier performance we generated a tweet clean text that took the mentions (@) and hyperlinks out of the tweet so that it was more readable and less noisy. This tweet clean text was the one used to train the classifiers which were applied to the tweet clean text.

Out of the 1500 manually labelled tweets, we reserved 10% to use as a blind set for model validation, and then the setup for training the classifier was 80% training and 20% validation. First, the training was done on the Classifiable feature and then, out of the tweets that were labelled classifiable, the rest of the classifications were trained. The weighted average F1 score of the training validation and against the blind dataset was above 0.80 in all the cases except in User and Interest area which was slightly lower.

These analyses were performed with Python 3.7 and using the libraries “pandas”, “numpy”, “json” and “ktrain”. Natural Language Processing is increasingly being used in healthcare research (http://dx.doi.org/10.5220/0010414508250832 (accessed on 14 December 2021)).

### 2.4. Ethical Issues

This study received the approval of the University of Alcala’s Research Ethics Committee and fulfils the research ethics principles stated in the Declaration of Helsinki (Seventh Revision, 2013). However, this research did not involve human subjects directly nor included any human intervention, as it used tweets that were publicly available. Nonetheless, we took special care not to reveal the users names and avoided including tweets that could disclose them.

### 2.5. Statistical Analysis

The frequency distribution (percentage) of tweets, retweets, and likes according to different characteristics of the tweet, such as the area of clinical interest, different non-medical contents, type of user or the mentioned antidepressant drug, were displayed across several figures and tables. Retweet-to-tweet and like-to-tweets ratios according to type of user, area of clinical interest and non-medical aspects were also calculated. The accuracy of the different tweet distributions obtained by these Multilingual Machine Learning models, is reflected by the weighted average F1 score (a combination of precision and recall, the closer to 1, the less possibility of classification error). These analyses were conducted with the software packages STATA v16 (StataCorp) and MS Excel.

## 3. Results

### 3.1. Sleep and Appetite/Weight Are the Most Common Areas of Clinical Interest among Tweets That Mention Antidepressants

In accordance with the criteria in the codebook, we included a total of 2880 tweets. Among them, 2089 were deemed classifiable. First, we classified tweets according to the specified areas of clinical interest (Table 1), 65.05% of tweets were categorized as undetermined. The rest, 730 out of 2089 (34.95%) made reference to these specific areas. We noticed a major difference in tweet number distribution among the included categories, sleep being the most predominant (20.87%). The next most frequently mentioned was appetite and weight (8.95%).

We also took into account those tweets that did not include medical aspects of antidepressants (341 tweets, 16.32%) and we classified them into three categories (commercial activities, help request/offer and drug trivialization) (Table 2). Interestingly, drug trivialization was the most frequent content found (228 tweets, 66.86%), whereas help requests/offers (4.99%) and those of a commercial nature (3.81%) were the least frequently posted.

We also considered other aspects. We found out that 39.5% of tweets were scientifically accurate (this percentage went up to 47.28% if we considered tweets with medical content) and that 16.6% mentioned the use of an antidepressant with a medical prescription. Finally, 13.87% discussed the use of antidepressants while suffering from a specific psychiatric disorder.

### 3.2. Patients and Their Friends and Family Are the Users That Discuss Antidepressants the Most in Twitter and Have More Interest in Medical Content about Antidepressants

We studied the type of user that posted the tweets; 55.1% of tweets were posted by users identified as patients and friends/relatives, 41.1% were considered interactions between Twitter users and only 3.5% were posted by caregivers.

**Figure 2:**
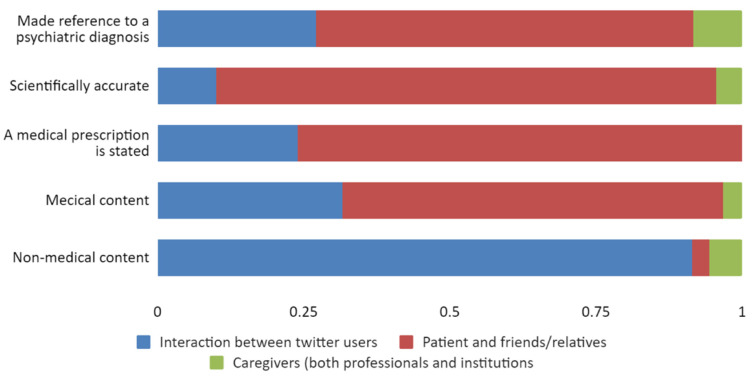
Proportion of type of user by considered aspects of tweets.

We then proceeded to study the type of content (medical/non-medical) per type of user. Of tweets with medical content, 65.2% were posted by patients and relatives, whereas users classified as interaction between Twitter users posted 91.5% of non-medical tweets. Tweets posted by caregivers were the least found in each and every category (Figure 2).

**Figure 3:**
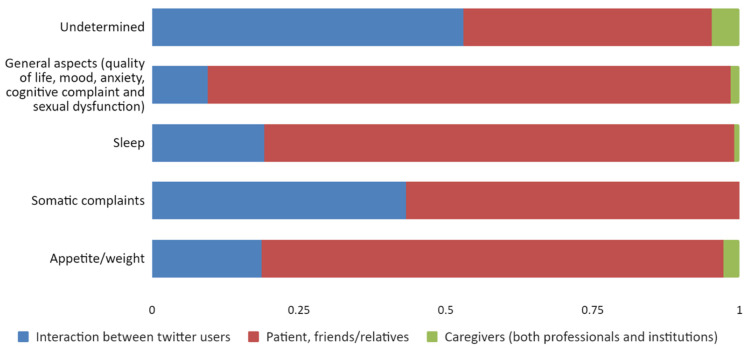
Proportion of type of user by area of clinical interest.

When looking more thoroughly into the connection between the different areas of clinical interest, we found important differences. Patients focused the subject of their tweets on specific clinical aspects, mainly sleep (80%) and appetite/weight (78.6%) (Figure 3).

**Figure 4:**
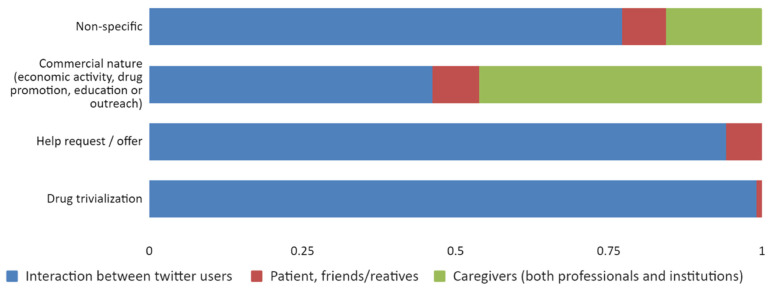
Proportion of type of user by non-medical content category.

On the contrary, interactions between Twitter users were responsible for 91.5% of non-medical content. Of the tweets that trivialized drugs, 99.1% came from this type of user alone. Finally, we would like to point out that almost all tweets posted by caregivers discussed non-medical content, and the most frequent subject in these tweets was of a commercial nature (46.2% of these tweets were posted by this type of user alone) (Figure 4).

### 3.3. Tweets That Rise Most Interest Are of a Commercial Nature and from Caregivers

**Figure 5:**
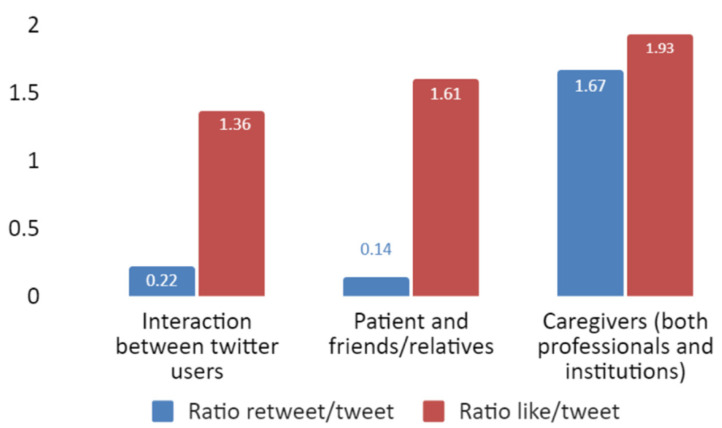
Retweet and like ratio (*Y*-axis) by type of user (*X*-axis).

We assessed the interest that tweets about antidepressants generated by quantifying retweets and likes per tweet. We found that the odds of a tweet being retweeted or liked were different depending on the different type of user. Tweets posted by caregivers had the highest ratio of retweets per tweet, 11.93 times higher than those posted in patient and friends/relatives. On the contrary, when we analysed the likelihood of receiving likes, we found that the ratio of likes per tweet is very similar between the different user type (Figure 5).

**Figure 6:**
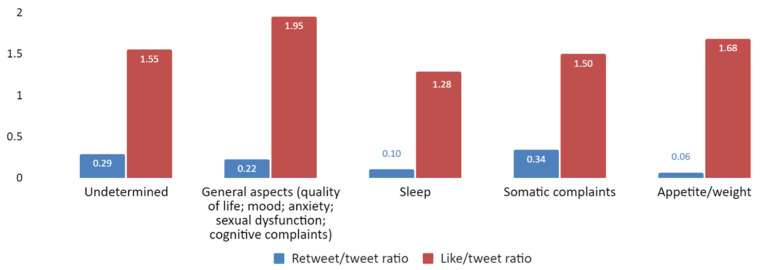
Retweet and like ratio (*Y*-axis) by area of clinical interest (*X*-axis).

Furthermore, we looked into the number of likes and retweets per area of clinical interest (Figure 6). Tweets about general aspects had the highest like ratio, followed by those about appetite/weight, although there was not a major difference with the rest of areas of interest. When considering retweets, differences between areas of interest were minimal.

**Figure 7:**
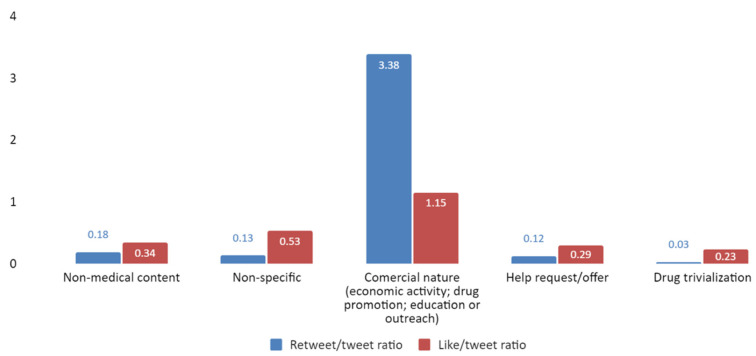
Retweet and like ratio (*Y*-axis) by non-medical content category (*X*-axis).

In addition, we studied the likelihood of a non-medical tweet being liked or retweeted. Tweets of a commercial nature were clearly more retweeted and liked than those that reference a help request/offer and those that trivialized drugs (Figure 7).

### 3.4. Tweet Frequency per Antidepressant Is Heterogeneous. Mirtazapine Is by a Large Difference the Most Mentioned Drug in Twitter

**Figure 8:**
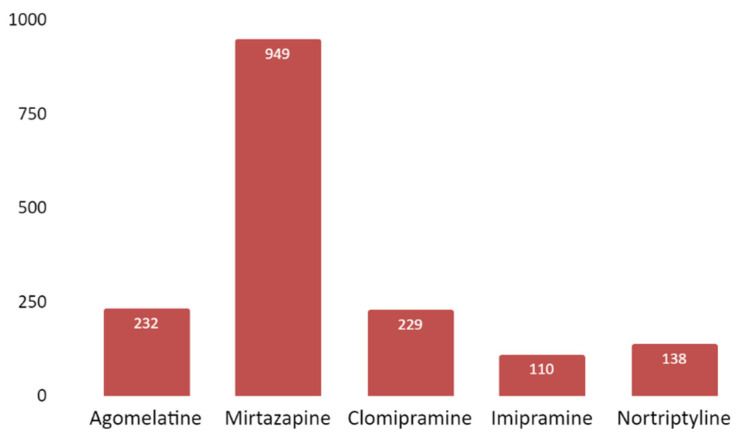
Number of tweets (*Y*-axis) per studied antidepressant (*X*-axis) (including both active ingredient and commercial name). Only antidepressants that were mentioned in at least 100 tweets are shown in the figure.

The number of mentions per drug followed a heterogeneous distribution pattern. It is noteworthy that 45.43% of tweets mentioned a single drug, mirtazapine. The next most mentioned drugs are agomelatine (11.11%), followed by clomipramine (10.96%), nortriptyline (6.61%) and imipramine (5.26%). The rest of the drugs were rarely mentioned (Figure 8).

## 4. Discussion

### 4.1. Major Findings

In this study, we found that in the Twitter community, the focus on antidepressants is mainly in the effects that these drugs may cause on certain health areas, specifically sleep and appetite/weight. Patients and their friends and relatives are the most frequent contributors in these matters, and, remarkably, their posts were very scientifically accurate.

Twitter users labelled as “Interaction between Twitter users” are responsible for the majority of non-medical tweets. Sadly, tweets trivializing several aspects of these drugs were the most common, whereas tweets requesting/offering help were the least common. Caregivers hardly have any presence in Twitter conversations about antidepressants. Nevertheless, their posts are the ones that rise more interest among Twitter users. Mirtazapine is the drug that appears the most and, although it is one of the most prescribed, others with a higher number of prescriptions are less mentioned in tweets.

Our findings show that patients and their friends and family are very active when talking about antidepressants and their effects, which contradicts previous findings from other studies, for example, in a previous study, only 13% of analysed tweets in bipolar disorder were about personal experiences [40]. In the case of another study about chemotherapy, tweets from patients and their friends/relatives added up to the 9.3% [41].

However, the fact that patients and their environment are the ones that generate more content about antidepressants is in line with previous research stating that Twitter benefits communication on mental health, including self-statements and support from people with similar experiences [42,43,44]. In other studies, Twitter users that claimed being depressed said that they considered themselves a burden for their friends and family, which, in turn, made them more prone to sharing their clinical situation in an online platform, given they were more anonymous that way [45,46,47]. Results from previous research allow us to comprehend the reason behind the high participation of patients and their environment in the tweets analysed in our study. These show that online health information is especially beneficial for patients that stay at home for long periods of time because of their illness, either immobilised or weakened by it [48]. This situation is frequent in the case of depression.

Our study shows that 50% of analysed tweets with medical content are scientifically accurate. Research conducted on other illnesses found the content of tweets to be highly rigorous. By comparison with previous research, the percentage of scientifically accurate tweets in our study is lower. However, in our study most of the tweets with medical content were posted by patients and their environment, and in interactions between Twitter users, whereas caregivers were not very active in this regard. In a study about health-related tweets, 53.2% were statements based on evidence whose veracity could be assessed [49]. Another study shows a scientific accuracy over 80% for all cases in tweets with medical content addressing psychosis and other illnesses [50]. Still, these results can be expected in these two studies taking into account the type of user: in them, more than half of the tweets were posted by health professionals and health companies.

On the contrary, caregivers, health professionals and institutions alike, are not very present in Twitter, which we consider worrisome. Previous research studied what the role of doctors in social media should be, especially psychiatrists, and what would their function and goals be [51]. These studies found that one of the main reasons behind the lack of participation of psychiatrists in the media was the difficulty to preserve patient confidentiality, that is of great importance in mental health. This would also explain why they are less present in social media than other medical specialities [52]. Beyond seeking to understand the reasons why caregivers are less present in social media such as Twitter, which we find alarming as we mentioned above, there is a lot of information about antidepressants in social media that is not being supervised by experts. The reason behind this concern is that previous studies show that scientific information available online can be used as a reliable and supportive source, benefiting patients, but also to spread false medical information, not based in scientific evidence. This misinformation can mislead, confuse and have a negative influence in patients. With antidepressants specifically, information not based in scientific evidence can have a negative influence in the patient’s attitude towards the treatment itself [53]. That is why we consider that caregivers should be more present in social media in order to improve assistance and defence, among others, of patients [51]. Along these lines, previous studies had similar findings on health information posted online [54].

We found that interest arisen per antidepressant among Twitter users was strikingly heterogeneous. This is congruent with previous research that showed that user interest in Twitter was significantly focused on certain areas of the study. In a study about rheumatological illnesses, user interest was focused on only two of the areas of the ones included in the research, and in another paper about side effects associated with glucocorticoids, interest was focused on two adverse effects [55,56].

Mirtazapine is the antidepressant that was mentioned in 45% of tweets. In the first place, we find it interesting that mirtazapine was the fourth most prescribed antidepressant in England in the year 2020, while in the USA, in 2020, it was not among the most prescribed [57,58]. However, antidepressants prescribed with more frequency than mirtazapine are less mentioned in tweets, which indicates that this is not a valuable criterion. We have tried to connect this bigger interest with differences in efficacy or with a higher withdrawal rate due to adverse effects. With regards to efficacy, it is not possible to consider differences in the efficacy of mirtazapine as opposed to other drugs, that would have explained the bigger interest awoken in Twitter by it. Even though there is some research that finds significant differences, these are questioned due to the bias that some trials may present. [15,59]. This leads to the conclusion that efficacy studies are insufficient to endorse a determinate clinical decision when recommending one antidepressant over another in cases of major depressive disorders [60]. We have not found conclusive results about user opinions on antidepressant efficacy. Furthermore, in relation to the withdrawal rate, even if some studies have found slight differences in antidepressant discontinuation due to side effects, there is no solid evidence of any difference between mirtazapine versus other antidepressants [61]. In any case, it is well known that mirtazapine has a unique profile of adverse effects, consisting of a higher probability of weight gain or appetite increase and drowsiness, as compared to other antidepressants [59].

If we take into account that our results show that sleep and appetite/weight are the specific clinical areas of interest that attract more attention among Twitter users, we can come to the conclusion that either mirtazapine is behind the mention of these areas, given these are among its main adverse effects, or that these areas themselves are of such interest to the general population that the effect that mirtazapine has on them makes it the most mentioned antidepressant. This last statement is supported by the findings of another study, in which insomnia and weight gain were the adverse effects most frequently mentioned in Twitter as side effects of glucocorticoids [56]. The fact that these are the most mentioned areas when discussing any drug supports the idea that these are areas of interest for Twitter users, both when the effects described are already known and established for a particular drug, as is the case of antidepressants and specifically mirtazapine, and when they have not yet been acknowledged by trials or doctors, as it happens with insomnia and glucocorticoids [56]. Both changes in weight and in circadian rhythms are common adverse effects described with antidepressants, among others [62,63]. In addition, as sleep disturbances and changes in appetite are frequent symptoms associated with depression, it is reasonable to conclude that their presence in tweets is due to a double reason: as symptoms to be tackled by antidepressants and as areas upon which antidepressants cause changes. It is remarkable that Twitter users do not mention core symptoms of depression as much, such as sad mood, somatic symptoms such as tiredness or cognitive symptoms such as concentration loss. Users have also shown less interest towards other frequent side effects, such as gastrointestinal discomfort, dry mouth or sexual dysfunction [64]. Taking all of this into account, we can conclude that sleep and gain or loss in weight are aspects that concern patients suffering from depression and that take antidepressants. Psychiatrists should consider this fact when prescribing antidepressants, examining these symptoms both in terms of finding out if they exist in the case itself or considering them when selecting the antidepressant for the patient’s treatment and follow-up. We can also support the conclusion already established by previous studies, that Twitter is an efficient source of information regarding pharmacovigilance [35,56,65,66,67]. Knowing what the general public considers of relevance about antidepressants is important for psychiatrists, but also information available online is important to patients, and can influence them when deciding about starting or continuing an antidepressant treatment [67]. As part of our findings, we insist on the need for psychiatrists and other mental health caregivers to increase their presence in Twitter and social media in general, with the goal of improving assistance and care of patients, both in consultation and in social media [51].

However, in the case of the study about glucocorticoids, there is a lack of connection between the frequency of side effects mentioned by patients in Twitter and general knowledge about them [56]. This is consistent with findings in another study about anti-obesity drugs, in which patients described adverse effects that were not identified by traditional means of inquiry [68,69]. In the case of antidepressants, Twitter users informed more frequently about adverse effects well known by professionals and very frequently discussed by patients in consultation. In previous research on the side effects of statins mentioned in Twitter, as it occurs with antidepressants, acknowledged adverse effects are the same as the ones already stated in other media (such as regulatory data or published papers) [28].

Negative, stigmatizing attitudes, including trivialization of illnesses or their treatment, have been previously studied. Analysis of social media platforms such as Twitter is a powerful tool that probes society’s opinion on chronic health conditions [70]. Compared to other traditional means of quantifying stigmatization, this type of analysis is less affected by social desirability [71]. Research conducted so far analyses beliefs and attitude towards illnesses in social media. Several types of diseases have been compared, such as heart diseases with cancer, and, specifically in mental illness, eating disorders, bipolar disorder and, more frequently, schizophrenia, that has been compared with depression or diabetes, where trivialization is not very likely [31,40,72,73,74,75,76,77]. This research found that stigmatizing attitudes towards mental illnesses were more frequent when considering bipolar disorder among mental illnesses in general, and more frequent in schizophrenia than in depression or in other medical conditions.

However, little research has been conducted about concerns and attitudes of social media users towards pharmacological treatment. A study was able to identify posts containing negative beliefs and opinions about statins, describing negative side effects and mistrust of medical care, but it did not find trivializing and stigmatising opinions. In recent years, research on Twitter has been conducted studying pharmacovigilance across social media: of psychotropic drugs, in particular, of other types of medication and a review study analysing feelings towards pharmacotherapy [35,56,78]. Our study explores uncharted territory, finding that antidepressants are also subject to ideas and negative feelings among Twitter users, resulting in mockery and drug trivialization. The percentage of antidepressant trivialization we found in our study is similar to that found in previous research about stigmatization and trivialization of mental illness, adding up to 10% of tweets and becoming practically unacceptable if we also included tweets of non-medical content (>65%). This data confirms that findings about antidepressants are in line with conclusions in other research about mental illness [50]. We also find relevant that the users posting trivializing tweets are not patients or their friends and relatives, so it is possible to conclude that stigma is fuelled by users that do not know the effects of these drugs closely. Furthermore, our findings reinforce the already established idea that illness is more stigmatizing if non-medically used rather than when it is directly alluded to [77]. Therefore, we conclude that negative consideration towards mental illness that, historically, has been present in other media, and has been also confirmed in social media, can be extrapolated to antidepressants.

### 4.2. Limitations

This study has some limitations. First, there is the difficulty of knowing the identity of the majority of users precisely, making it impossible to generalise the findings of this study to the wider population. Twitter users are usually younger, have more education, and have higher incomes than the overall population, so it may bias the results [79]. In addition, the sample size was not big enough to gather significant information on certain clinical areas. Furthermore, qualitative analysis of the tweets’ content has an inherent degree of subjectivity, as information about context can be limited, and also due to codebook design, that may result in the omission of certain aspects relevant to users, leaving them out of the analysis. Expert subjectivity can be a bigger limitation when specifically coding trivializing content, in which emotional tone or double entendre are a very important aspect. However, these effects were reduced to a minimum by carefully designing the codebook and training the experts thoroughly prior to the start of the codification, experts who were also psychiatrists. We would also like to highlight the fact that, limitations aside, our methodology is consistent with previous medical research on Twitter.

## 5. Conclusions

This study shows that Twitter users that take antidepressants, or their friends and family, use social media to share medical information about antidepressants. However, other users that do not talk about antidepressants from a personal or close experience, frequently do so in a stigmatising manner, by trivializing them. Our study also brings to light the scarce presence of caregivers in Twitter. This may be an opportunity for mental health caregivers to increase their numbers in social media in order to comprehend what patients and their environment think of drugs, allowing these professionals a better choice and supervision of antidepressants in consultation. They can also potentially reduce the stigma that antidepressants carry, by participating in trivializing conversations about drugs, and clarifying aspects that could otherwise influence patients and their antidepressant treatment negatively.

## Figures and Tables

**Table 1:** Number and percentage of tweets by area of clinical interest.

	Tweets	
	*n*	%
Area of clinical interest	2089	100.00%
Undetermined	1359	65.06%
General aspects (quality of life; mood; anxiety; sexual dysfunction; cognitive complaints)	63	3.02%
Sleep	436	20.87%
Somatic complaints	44	2.11%
Appetite/weight	187	8.95%

**Table 2:** Number and percentage of tweets by non-medical category.

	Tweets	
	*n*	%
Non-medical content	341	100.00%
Non-specific	83	24.34%
Commercial nature (economic activity; drug promotion; education or outreach)	13	3.81%
Help request/offer	17	4.99%
Drug trivialization	228	66.86%

## Supplementary Materials

The following supporting information can be downloaded at: https://www.mdpi.com/article/10.3390/jpm12020155/s1, File S1: Category, definitions and examples of classification.
