# Supplementary material for: Areas of Interest and Social Consideration of Antidepressants on English Tweets: A Natural Language Processing Classification Study
Source: J Pers Med. 2022 Jan 25;12(2):155. doi: 10.3390/jpm12020155 (PMC8879287; doi:10.3390/jpm12020155)
Supplement: Supplementary file 1 [file jpm-12-00155-s001.zip › jpm-1532648-Supplementary.pdf]

File S1. Category, definitions and examples of classification. Usernames and personal names in the tweets have been removed (they were replaced by XXX).

Category	Definition of category	Example tweet
**Area of interest**		
**General aspects**	The tweet refers to mood or anxiety - related symptoms, cognitive complaints, sexual dysfunctions or subjective perception of overall well-being.	*mechanism is inhibition of reuptake of sertonin like SSRIs .. That made it applicable for depressed status and men who addict Anafranil and dapoxetin and stuff like that !! https://t.co/bIWSAkRqzJ*<br><br>*@xxx I regularly grapple with anxiety and make forays into depression. I've been taking Valdoxan on and off to help manage things for about 5 years now.*<br><br>*@xxx try remeron.*<br>*45 mg later, i have difficulty counting to one.*<br><br>*@xxx Prozac and REMERON, which was the best ad I took before finding out I'm bipolar lol, had really few sexual side effects*<br><br>*@Dwayne__Tuck I was the same until my psych added Remeron (mirtazapine) to the cocktail. Her drug interaction manual calls it "California Rocket Fuel" (Google it). Been AWESOME ever since!*
**Sleep**	The antidepressants is related to some aspect of sleep, either qualitatively or quantitatively	*remeron has me exhausted today. i took it at 9pm last night, slept for nearly 10 hours, and i still feel drowsy. i'm hoping this side effect will go away once my body acclimates to the chemicals.* [illegible]<br><br>*@xxx Remeron...saved my life. They say it's an anti-depressant but a low dose is an amazing sleep aid. I tried everything, literally everything, this is the only thing that works. Makes ya gain a bit of weight though*
**Weight/appetite**	The tweet refers to weight or appetite as a drug-related area of concern	*Of course, a side effect to that increased appetite is weight gain. I've put on over 30 pounds since I switched to Remeron without trying or anything. It's real, and honestly doesn't bother me one bit.*<br><br>*@xxx The first medication I tried (remeron) did sort of turn me into a zombie, tho. I never improved and just slept tons and weirdly gained a lot of weight in a short period of time. I never had trouble like that with Paxil, though.;*

**Somatic complaints**	Somatic aspects related to the antidepressant is the main topic of tweet	*started on Sertraline (Zoloft), and I noticed my mood lifting, but it made me sweat loads which added to my anxiety and was just generally not fun! Mirtazapine (Remeron) was next and it worked, but it made me dizzy. Being spaced out was nice but not practical.*<br><br>*@xxx Good luck! I recently came off Remeron (Mirtazapine) cold turkey. The 1st week was horrible, brain zaps, fever, upset stomach, etc. Then increased anxiety and insomnia. It's now been 5 weeks and I'm sleeping again and feeling normal. It's been awful. No one warns you about this.*
**Reference to psychiatric diagnosis**	Psychiatric diagnosis related to the antipsychotic drug appears in the tweet	*@xxx Cocktail of Remeron/Effexor/Abilify/Prazosin for Treatment Resistant Major Depressive Disorder here! No shame! Meds, CBT, counselling and 2 great Drs got me to where I can experience joy again! #SickNotWeak*<br><br>*@xxx prozac, lexapro and anafranil to be specific. for severe OCD. it's real and debilitating but i'm just tryna thrive*
**Scientifically accurate**	The information in the tweet regarding the antidepressant drug is consistent with current scientific literature	*@xxx I mean mirtazapine/remeron 7mg is usually prescrbed for insomnia and the higher doses for depression but youd have to rly speak to ur doc about that (plus you'd have to check interaction with other drugs you do/take)*<br><br>*medication update:*<br>*it's been 2 and a half weeks on remeron and it's really started to level out a bit, it's not making me as drowsy as it did the first week i took it, and my appetite has increased a lot (which i actually hate). i am about to eat alfredo chicken at 10pm.*
**Use according to medical prescription**	The information provided in the tweet shows that the drug is being taken according to a doctor's prescription and not as a form of self-medication	*Generalized anxiety disorder & depression, not diagnosed until my mid 20s but with symptoms since 9 y/o, I'm currently on meds (Imipramina/Alprazolam) currently on my 4yr, if not for them I couldn't have the amazing life I now have, I see a psychiatrist once a yr for a follow up https://t.co/Lknz8JoFdN*

		I talked to a psych today. Adding lamictal to my Effexor/remeron regimen. Cheers to mental stability!

**Non-medical content**		
**Comercial nature**	The tweet refers to economic activity, drug promotion or education or outreach	*@xxx I was prescribed Valdoxan, and it worked well but I just couldn't keep up with the cost.*<br><br>*Fellow valdoxan people where we getting it cheapest now? https://t.co/77M7fLqky7 #Sydney*<br><br>*@xxx are Anafranil still distributed in Indonesia?*<br><br>*A recall for the generic depression medication Remeron has been issued due to a labeling error that could increase the risk of overdose. https://t.co/jzSoAJOV8C*<br><br>*Anafranil Oral : Uses, Side Effects, Interactions https://t.co/q0tb4kcPbq https://t.co/76gJ2OWQUS*
**Help request/offer**	The tweet is primarily dedicated to practical help, such as getting a drug, not just medical advice that would qualify as medical content	*@mickey_mcvay @UghAsIf_1980 @PolitiBunny @davidmweissman @JoyAnnReid 2020 is gonna be so hard to take. Seems you need some state maintained psychiatric care. Aricept perhaps, maybe Remeron. Either way you need to seek help. Your TDS is out of control.*<br><br>*if anyone has any spare/leftover/sample valdoxan, please hmu, each pill is $2*
**Drug trivialization**	The tweet refers to the drug in a way that detracts from its value	*You know what this night needs? Zyprexa, Remeron, Trazodone, melatonin, vodka, dab pen…*<br><br>*if i was a psych and i ever thought my client was annoying i would simply prescribe them remeron*
